# Peripheral Precocious Puberty of Ovarian Origin in a Series of 18 Girls: Exome Study Finds Variants in Genes Responsible for Hypogonadotropic Hypogonadism

**DOI:** 10.3389/fped.2021.641397

**Published:** 2021-05-07

**Authors:** Raja Brauner, Joelle Bignon-Topalovic, Anu Bashamboo, Ken McElreavey

**Affiliations:** ^1^Hôpital Fondation Adolphe de Rothschild and Université Paris Descartes, Paris, France; ^2^Human Developmental Genetics Unit, Institut Pasteur, Paris, France

**Keywords:** genetic variant, hypogonadotropic hypogonadism, ovarian cyst, precocious puberty, prenatal ovarian cyst

## Abstract

**Background:** Peripheral precocious puberty of ovarian origin is a very rare condition compared to central form. It may be associated with an isolated ovarian cyst (OC). The causes of OC in otherwise healthy prepubertal girls is currently unknown.

**Methods:** Exome sequencing was performed on a cohort of 18 unrelated girls presenting with prenatal and/or prepubertal OC at pelvic ultrasonography. The presenting symptom was prenatal OC in 5, breast development in 7 (with vaginal bleeding in 3) and isolated vaginal bleeding in 6. All had OC ≥ 10 mm. The girls had no other anomalies. Four patients had a familial history of ovarian anomalies and/or infertility.

**Results:** In 9 girls (50%), candidate or known pathogenic variants were identified in genes associated with syndromic and non-syndromic forms of hypogonadotropic hypogonadism including *PNPLA6, SEMA3A, TACR3, PROK2, KDM6A, KMT2D, OFD1, GNRH1, GNRHR, GLI3, INSR, CHD7, CDON, RNF216, PROKR2, GLI3, LEPR*. Basal plasma concentrations of gonadotropins were undetectable and did not increase after gonadotropin-releasing hormone test in 3 of them whilst 5 had prepubertal values. The plasma estradiol concentrations were prepubertal in 6 girls, high (576 pmol/L) in one and not evaluated in 2 of them.

**Conclusions:** In the first study reporting exome sequencing in prepubertal OC, half of the patients with OC carry either previously reported pathogenic variants or potentially pathogenic variants in genes known to be associated with isolated or syndromic forms of congenital hypogonadotropic hypogonadism. Functional studies and studies of other cohorts are recommended to establish the causality of these variants.

## Introduction

Precocious puberty (PP) in girls is defined by the development of sexual characteristics (development of breast, pubic or axillary hair and/or menstrual bleeding) before 8 years-of-age (1). There are two major categories of PP, the true or central and pseudo or peripheral PP. Central PP is associated with the premature activation of the hypothalamic-pituitary-gonadal axis (1, 2). Isosexual peripheral PP is characterized by the development of breast and/or menstrual bleeding due to primary estradiol secretion originating from either the ovaries or from adrenals in the absence of hypothalamic-pituitary-gonadal axis activation. Central and peripheral PP can be distinguished by measuring basal and gonadotropin releasing hormone (GnRH)-stimulated luteinizing hormone (LH) and follicle stimulating hormone (FSH) peaks concentrations. These concentrations increase in central forms (3), whilst they are low and do not increase in peripheral PP.

Peripheral PP of ovarian origin is a very rare condition compared to central form. It may be due to granulosa cell tumor or may be one symptom of the McCune-Albright syndrome (4). It may also be associated with an isolated ovarian cyst (OC) (4), where it is often transient and recurrent (5). The causes of OC in otherwise healthy prepubertal girls is currently unknown.

In a previous study, we analyzed an exceptional series of 11 unrelated girls who presented with an isolated OC seen at the pelvic ultrasonography performed *in utero* and/or in a prepubertal age (6). In 4 cases there was a familial history of ovarian anomalies and/or infertility. We have tested the hypothesis that mutations in the *GNAS1, LHCGR, FSHR, StAR, NR5A1, DMRT4*, and *NOBOX* could contribute to the phenotype, but we found no potentially pathogenic variants in these genes. Since that study, we have seen 8 other girls with OC.

The objective of this study was to assess by exome sequencing potential pathogenic variants in 18 girls with OC diagnosed prenatally and/or before 8 years of age. Exome sequencing revealed potentially pathogenic variants in genes known to be associated with either isolated or syndromic congenital hypogonadotropic hypogonadism (HH) in 9 of the girls.

## Materials and Methods

### Setting

This retrospective single center study was performed on 18 unrelated girls with OC measuring ≥10 mm diagnosed prenatally and/or before 8 years of age, with the exception in two girls aged 8.8 and 9.2 years, first seen between 1996 and 2017 (21 years) by a senior pediatric endocrinologist (R.B.) in a university pediatric hospital. Among the 11 girls previously reported (6), one (case 7 in ref 6) was excluded because her DNA sample was unavailable. As the data of these had been previously detailed, only the data of the girls carrying mutation are detailed in the present report.

### Participants

McCune-Albright syndrome was excluded by clinical evaluation, complete skeletal radiographic examination (*n* = 11, not performed in 4 with prenatal OC and in cases 9, 11 and 17, Table 1). A mutation of arginine 201 in the Gs protein in blood was excluded in cases 1, 2, 4, 6, 7, 8, 10, 13–15, and 18 including in the OC liquid collected at surgery in case 13 (6).

**Table 1:** Clinical, biological, ultrasonographic data, and evolution of 18 girls with prepubertal ovarian cyst.

**Case**	**First symptom**		**First evaluation**							**Evolution**	**Age (yr) at last evaluation**	**Gene with potentially pathogenic variant(s)**
	**Age (yr)**	**Type**	**Age (yr)**	**Tanner Stage**	**GnRH stimulation test**		**Estradiol (pmol/L)**	**Pelvic ultrasonography**				
					**LH basal, peak (IU/L)**	**FSH basal, peak (IU/L)**		**Ovarian cyst (mm)**	**Uterus length (mm)**			
1	Prenatal	OC	1.8	B3P1	0.6–5.2	2–30.5	<7.3	Ovaries not seen	Normal	R OC 25 mm, not found at birth	2	*PNPLA6*
2	Prenatal	OC	1.3	B2P1	1–7.2	2.7–34.5	<37	Normal R ovary	25	L ovariectomy	2.4	*SEMA3A*
3	Prenatal	OC	9.7	B1P1	ND	ND	ND	Normal R ovary	20	L ovariectomy	13	
4	Prenatal	OC	1.7	B1P1	0.9–2.2	1.8–23.5	22	Normal ovaries	27	R OC 48 mm, progressively disappeared afer birth	1.7	
5	Prenatal	OC	6.7	B1P2	ND	ND	ND	ND	ND	L ovariectomy	21.4^***^	*TACR3/PROK2*
6	0.1	B2 M	0.8	B2P1	1–7.2	7–28	<37	Ovaries not seen	30	Recurrent M	21^***^	
7	0.1	B2 M	2.1	B3P1	0.5–4.3	1.6–14	ND	Normal ovaries^*^	ND	Recurrent M	23.5^***^	
8	0.8	M	0.9	B2P1	<0.05	<0.05	99	L 42	increased	L ovariectomy	0.9	
9	1.2	M	1.2	B1P2	<0.4–6.2	4.1–35	22	Normal ovaries	Normal	?	1.2	*KDM6A/KMT2D*
10	1.5	M	2.2	B2P1	<0.4–0.6	<0.4–0.8	33	L 17	36	Recurrent M, CA treatment	10	
11	5.1	B3	5.1	B3P1	<0.1– <0.1	<0.2–0.3	1,163	R 51	53	Regression OC and E2 <10 pg/ml after 1 mo	5.2	
12	5.6	B2 P2	5.7	B3P2	<0.4– <0.4	<0.4– <0.4	918	L 60	53	CA treatment	13.5^***^	
13	5.8	B2	6.6	B3P1	<0.2–0.74	<0.2–0.5	<37	Polycystic ovaries	34	Ovariopexy	9.5	*OFD1*
14	6.5	M	6.5	B4P2	<0.4– <0.4	<0.4–0.4	14.7	L 10	48	?	6.5	*GNRH1/GNRHR/GLI3/INSR*
15	6.6	B2	6.7	B3P1	<0.4– <0.4	<0.4– <0.4	576	L 41	58	CA treatment	13^***^	*CHD7/CDON*
16	7.8	B2 M	7.8	B3P1	<0.1– <0.1	<0.1– <0.1	594	R 33	71	?	7.8	
17	8.8	M	8.9	B2P3	<0.4–3.2	1.2–11	ND	Polycystic ovaries	34	?	8.9	*RNF216/PROKR2*
18	9.2	M	9.2	B1P1	<0.2–1.2	0.53–5	<37	Normal ovaries^**^	25	Recurrent M	13^***^	*GLI3/LEPR*

* *Ovarian cyst of 20 mm at 5 years;*

** *blood effusion in the Douglas cul-de-sac*,

*** *regularly menstruated. OC, ovarian cyst; B, breast; P, pubic hair; M, vaginal bleeding; L, left; R, right; ND, not determined; CA, cyproterone acetate*.

**Table 2:** Variants in genes associated with both non-syndromic and syndromic HH identified in 9 girls with ovarian cyst.

**Case**	**Gene**	**Variant**	**Minor allelic frequency**	**Predicted effect on protein**			**ClinVar ID; clinical significance; ACMG classification**
				**SIFT**	**PolyPhen2**	**REVEL**	
1	*PNPLA6*	NM_001166111:c.73A>T: p.K25X	Novel	–	–	–	–;–; VUS
2	*SEMA3A*	NM_006080:c.57A>C:p.R19S (rs139951141)	African 0.0018	Tolerated (0.76)	Benign (0.001)	0.238	–;–;VUS
5	*TACR3*	NM_001059:c.824>A:p.W275X^a^ (rs144292455)	European 0.00038	–	–	–	66084; Pathogenic; pathogenic
	*PROK2*	NM_001126128:c.301C>T:p.R101W (rs144953748)	South Asian 0.00049	Deleterious (0)	Probably_damaging (0.986)	0.853	–;–;VUS
9	*KDM6A*	NM_021140:c.139G>A:p.E47K	Novel	Tolerated (0.08)	Probably_damaging (0.953)	0.213	–;–;VUS
	*KMT2D*	NM_003482:c.1298C>T:p.P433L (rs759548512)	African 0.00011	Deleterious (0.05)	Benign (0.047)	0.277	–;–;VUS
13	*OFD1*	NM_003611:c.950A>G:p.Q317R	Novel	Deleterious (0.02)	Probably_damaging (0.951)	0.348	–;–;VUS
	*OFD1*	NM_003611:c.951G>T:p.Q317H	Novel	Tolerated (0.19)	Benign (0.337)	0.351	–;–;VUS
14	*GNRH1*	NM_000825:c.153G>C:p.E51D (rs35542850)	South Asian 0.00224	Tolerated (0.12)	Probably_damaging (0.991)	0.124	–;–;VUS
	*GNRHR*	NM_000406:c.317A>G:p.Q106R^a^ (rs104893836)	European 0.0039	Deleterious (0)	Probably_damaging (0.992)	0.606	16023; Pathogenic, likely pathogenic; likely pathogenic
	*GLI3*	NM_000168:c.2044A>G:p.T682A (rs760184006)	European 0.000045	Tolerated (0.27)	Benign (0.015)	0.111	–;–;VUS
	*INSR*	NM_000208:c.3218A>G:p.N1073S (rs748022366)	African 0.000097	Deleterious (0)	Benign (0.308)	0.482	–;–;VUS
15	*CHD7*	NM_017780:c.8416C>G:p.L2806V (rs45521933)	African 0.006697	Tolerated (0.31)	Possibly_damaging (0.458)	0.126	95814; benign, likely benign; likely benign
	*CDON*	NM_016952:c.1109T>C:p.V370A	Novel	Tolerated (0.08)	Possibly_damaging (0.716)	0.308	–;–;VUS
17	*RNF216*	NM_207111:c.785G>A:p.R262H (rs191573023)	Latino 0.00086	Tolerated (0.06)	Benign (0.169)	0.016	–;–;VUS
	*PROKR2*	NM_144773:c.253C>T:p.R85C^a^ (rs141090506)	Latino 0.001	Deleterious (0)	Probably_damaging (1)	0.422	156562; Pathogenic, likely pathogenic; likely pathogenic
18	*GLI3*	NM_000168:c.3935T>G:p.M1312R (rs199875457)	Latino 0.001101	Deleterious (0.01)	Benign (0)	0.126	283130; likely benign, uncertain significance; VUS
	*LEPR*	NM_002303:c.2728C>T:p.P910S	Novel	Deleterious (0)	Probably_damaging (0.999)	0.598	–;–;VUS

*The population showing the highest MAF from GnomAD is indicated*.

a *Previously reported as a cause of HH*.

β human chorionic gonadotropins and α-fetoprotein were measured in 11 girls at the initial evaluation (except cases 2, 3, 5–7, 14, and 18) and remained normal during the follow-up in cases 10 and 13. An adrenal disease was excluded, mainly in those with pubic hair development, by measuring basal plasma concentrations of 17-hydroxyprogesterone (*n* = 6 of whom 3 also after adrenocorticotropic hormone test), testosterone (*n* = 9), delta 4 androstenedione (*n* = 6) and dehydroepiandrosterone sulfate (*n* = 3). All these concentrations were normal. In addition basal plasma concentrations of inhibin B and anti-Müllerian hormone were measured in 9 and 4 girls, respectively. A hypothalamic-pituitary lesion was excluded by magnetic resonance imaging at the first evaluation in those with gonadotropins increase after the GnRH test (*n* = 7, not performed in the 5 with prenatal OC).

### Methods

The clinical biological evaluation was conducted as previously described (6). Pubertal changes were rated according to Marshall and Tanner (7). The hypothalamic-pituitary-ovarian axis was evaluated by measuring basal and GnRH-stimulated LH and FSH peaks and the plasma estradiol concentrations in all but 2 girls seen after prenatal OC (cases 3 and 5). For the GnRH test, we used Gonadorelin (Relefact, Ferring SAS, 100 μg/m^2^, maximum 100 μg), with serum samples collected at 0, 30, 60, and 90 min after the injection. LH and FSH concentrations were measured using a two-site monoclonal immunoradiometric assay (LH-Coatria and FSH-Coatria; bioMerieux, SA, Marcy-l'Etoile, France). The within-assay coefficients of variation (CV) ranged from 3 to 7% for LH and 4.5% for FSH. The between-assay was 11.4 and 7.8%, respectively. Estradiol was extracted with ether and measured using a radioimmunoassay (Estradiol-2; Sorin Biomedica, Antony, France). The within- and between-assay CV were 4 and 7% for estradiol. Serum LH, FSH and estradiol concentrations were measured using various radioimmunoassays during the study period. Each new assay for a given hormone was cross-correlated with the previous method to ensure comparable results for a given parameter throughout the study period. The sensitivity of the methods to measure plasma estradiol varied and this explains the different cut-offs for the low values (Table 1). The values considered to be prepubertal were: uterus length of <35 mm, LH/FSH peaks ratio after GnRH test <0.66 (3), and plasma estradiol concentrations <15 pg/ml (55 pmol/l).

### Genetic Analyses

Whole exome sequencing and exon enrichment was performed as described elsewhere (8). We used Agilent SureSelect Human All Exon V4. Paired-end sequencing was performed on the Illumina HiSeq2000 platform with an average sequencing coverage of ×50. Read files were generated from the sequencing platform via the manufacturer's proprietary software. Burrows-Wheeler Aligner (BWA) was utilized to map the paired-end clean reads to the human reference genome (hg38, http://hgdownload.cse.ucsc.edu/goldenPath/hg38/bigZips/analysisSet/hg38.analysisSet.2bit). SAMtools was used for sorting the BAM files, and Picard was utilized to mark duplicate reads. Local realignment of the mapped reads around potential insertion/deletion (indel) sites was carried out with the GATK version 1.6. SNP and indel variants were called using the GATK Unified Genotyper for each sample. SNP novelty was determined against dbSNP138. The fraction of exome targets with >20× coverage was >92% and for >10× coverage was >98%. Datasets were filtered for novel or rare (MAF < 0.01) variants. Novel and rare variants were analyzed by a range of web-based bioinformatics tools using the EnsEMBL SNP Effect Predictor (http://www.ensembl.org/homosapiens/userdata/uploadvariations). All variants were screened manually against the Human Gene Mutation Database Professional (Biobase) (http://www.biobase-international.com/product/hgmd). *In silico* analysis was performed to determine the potential pathogenicity of the variants using Polyphen (http://genetics.bwh.harvard.edu/pph), and SIFT (http://sift.jcvi.org/www/SIFT_chr_coords_submit.html) online tools that predict the effect of human mutations on protein function. We focused our analyses on non-synonymous coding, nonsense, and splice site variants, filtering out all known common variations contained in dbSNP (build 138) (www.ncbi.nlm.nih.gov/projects/SNP/), the 1000 Genomes Project (http://www.1000genomes.org/) and in the gnomAD database (http://gnomad.broadinstitute.org/). Variants with an MAF > 0.01 in any specific subpopulation were excluded. The remaining rare or novel variants were confirmed by visual examination using the IGV browser and potential pathogenic variants were confirmed by Sanger sequencing.

## Results and Discussion

### Clinical, Biological, and Imaging Data

The patients were classified according to their age at the first symptom (Table 1). The presenting symptom was prenatal OC in 5 girls, breast development in 7 girls (associated with vaginal bleeding in 3) and isolated vaginal bleeding in 6 girls. All girls had either a large OC (≥10 mm) or polycystic ovaries (cases 13 and 17). In case 9, the ovaries were described as normal on an ultrasonography performed after vaginal bleeding at 1.2 years. She was included in the series because of the vaginal bleeding at 1.2 years which may have resulted from estradiol withdrawal after cyst resolution.

Overall the basal plasma concentrations of LH and FSH were undetectable and did not increase after GnRH test in 8 girls (case 8 and cases 10 to 16, Table 1). Five of these girls had high plasma concentrations of estradiol, while the 3 others had prepubertal concentrations. In 8 other girls the LH and FSH concentrations increased after GnRH test with normal prepubertal responses and plasma estradiol concentrations. Of these 8 girls, 6 were <2 years of age and had a predominant FSH increase, which may be due to the mini-puberty. Gonadotropin concentrations were not evaluated in 2 girls, who were seen after prenatal OC.

There was no history of personal or familial anosmia, nor consanguinity. Four patients had a familial history of ovarian anomalies and/or infertility (cases 4, 6, 13, 15, Figure 1 in 6). Of these, we detailed cases 13 and 15 with potentially pathogenic variants (see below). No potentially pathogenic variants were found in the two other familial forms of OC: in case 4: mother with an orange-sized OC that was removed surgically in emergency at the age of 10 years; in case 6: four paternal aunts had vaginal bleeding and all were reported as infertile; in addition, both the mother and maternal grandmother also reported to have menometrorragia. Hysterectomy was performed in the mother at 42 years because of uterus fibroma diagnosed at 30 years.

### Follow-Up

Four of the girls were seen when vaginal bleeding occurred (cases 9, 14, 16, and 17) and they were followed elsewhere (Table 1). In 5 girls with the antenatal diagnosis, the OC regressed spontaneously in 2 of the girls (absent at birth in case 1; progressively disappeared in case 4) or persisted leading to left ovariectomy in the 3 other girls. Surgery was also performed in case 8 because of a suspicion of an ovarian tumor due to a plasma α-fetoprotein concentration of 44 ng/ml at 10 months and the heterogeneous aspect of the lesion on imaging; the histology showed luteal OC. Surgery was also performed in case 13 for histological examination and to prevent torsion of voluminous ovaries. Three patients were treated with Cyproterone Acetate because of the recurrence of vaginal bleeding, marked estrogenisation and/or large OC (Table 1). In the untreated girls, the vaginal bleeding was recurrent in 4 girls, among them 3 are regularly menstruated at the last evaluation.

### Genetic Analyses

Initial genetic analysis focused on case 5, which revealed a p.W275X variant in the *TACR3* gene, a well-characterized pathogenic variant known to cause HH. Both biallelic *TACR3* variants, as well as variants in a single *TACR3* allele (including p.W275X) are associated with HH (9, 10). In the latter situation, the phenotype is considered to be due to the present of an independent mutation in a different HH gene. A further examination of the exome dataset in this girl revealed a rare heterozygous missense variant p.R101W in the *PROK2* gene (Table 2). Variants in the *PROK2* gene are associated with autosomal dominant HH and the p.R101W variant, located in the second transmembrane domain of the protein, is predicted to the highly damaging to the protein by multiple *in silico* tools (11). The genotype of this girl is consistent with HH. Prenatal OC was diagnosed and punctured *in utero*. The cyst persisted postnatally resulting in a left ovariectomy and annexectomy at 5 days. She was evaluated because of pubic hair development at 6.3 years and at that time no adrenal anomalies were found. She menstruated at 12.7 years, and then regularly until the last evaluation at 21.4 years. Unfortunately, data on the gonadotropin concentrations was unavailable for this girl and it is unknown if there is gonadotropin deficiency due to the presence of both pathogenic TAC3R and PROK2 variants.

These data suggested that there may be a relationship between OC genetic variants in genes known to cause HH. The basal and GnRH stimulated LH and FSH concentrations of 8 girls of the cohort also suggested that this may be the case. Therefore, all datasets were screened for rare/novel variants associated with isolated or syndromic forms of HH.

In case 1, a novel heterozygous nonsense variant in the *PNPLA6* gene was observed. She was seen at 1.8 years because of persistent breast development, which was noted at birth after a history of prenatal OC. Gonadotropins increase after GnRH test and plasma estradiol concentration were prepubertal. Biallelic *PNPLA6* variants are associated with neurodegeneration and impaired LH release from pituitary gonadotropes, leading to normosmic HH. *PNPLA6* encodes an enzyme that catalyzes the de-esterification of membrane phosphatidylcholine into fatty acids and glycerophosphocholine and recessive variants cause Gordon Holmes syndrome characterized by cerebellar ataxia/atrophy and normosmic HH (12) and Boucher-Neuhauser syndrome which in addition includes chorioretinal dystrophy (13). The neurological characteristics become gradually obvious during mostly the 2nd or 3rd decade (13).

Case 2 carries a rare heterozygous *SEMA3A* gene variant p.R19S. She had a prenatal right OC of 28 mm seen at 33 weeks of gestational age, increasing to 42 mm at 26 days after birth leading to right ovariectomy and annexectomy. She was seen at 1.3 years because of persistent breast development, noted at 6 months and recently increasing. She had no OC at ultrasonography, and had prepubertal gonadotropins increase after GnRH test and plasma estradiol concentration. The last evaluation, at 2.4 years, indicated persistent breast development. Heterozygous *SEMA3A* variants are associated with an autosomal dominant form of HH-16 with or without anosmia (HH16) (14, 15).

Non-synonymous rare or novel variants in the *KDM6A* and *KMT2D* genes were observed in case 9. She was seen at 1.2 years because of vaginal bleeding associated with pubic hair development. The gonadotropins increase after GnRH test, plasma estradiol concentration and uterus at ultrasonography were prepubertal. She had no other personal history, nor follow-up after this episode. *KDM6A* and *KMT2D* variants are associated with Kabuki syndrome (16). *KDM6A* variants are associated with an X-linked dominant form, whereas *KMT2D* variants are associated with an autosomal dominant form. Classic Kabuki syndrome consists of intellectual deficiency, postnatal dwarfism, typical facies, radiographic abnormalities of the vertebrae, hands, and hip joints, and recurrent otitis media in infancy. Devriendt et al. reported a hepatic cystic mass, extending into the pelvis, diagnosed prenatally in a girl with Kabuki syndrome (17). It was removed and pathological examination showed a typical follicular cyst. Multiple additional small cysts were present in both ovaries. Interestingly, premature breast development, without details on whether the origin was central or peripheral, is a common finding in patients with Kabuki syndrome (18), and more frequently in those carrying *KMT2D* pathogenic variants than in those without (19). Delayed puberty has also reported in this syndrome (20).

In case 13, there is a family history of OC. Her mother had an ovarian dermoid cyst at 25 years, leading to an ovariectomy; her mother's aunt was infertile, due to an undetermined ovarian trouble, after a menarche at the age of 10 years; her grand-mother's cousin underwent ovariectomy for an OC (Figure 1 case 9 of ref 6). She also has congenital strabismus. She was referred for breast development at 5.8 years. Basal plasma concentrations of LH and FSH were low and did not increase after GnRH test, while the estradiol concentration was prepubertal. Ultrasonography showed polycystic ovaries. During the initial follow-up, ultrasonography showed no modification. She was not treated because breast development and bone age did not progress. At 9.3 years, her Tanner stage progressed to B4, and ovaries size was increased, with a larger diameter of 60 and 70 mm and many cysts, measuring 8–12 mm. The plasma concentrations were 381 pmol/l for AMH and 94 pg/ml for inhibin B. At 9.4 years, ovariopexy and multiple ovarian biopsies were performed: macroscopically, both ovary diameters were more than 100 mm, and there were multiple cortical cysts, with no sign of malignancy. Plasma estradiol concentration in the cyst fluid was 1,945 pmol/l. After surgery, she has unexplained and persistent hypertension. At the last evaluation (12.5 years), she had complete breast and pubic hair developments, uterus at 55 × 42 × 16 mm, but had not yet menstruated. The ovaries size decreased to 65 and 47 mm and multiple small cortical follicles with an echogenic and large stroma. This patient carries compound heterozygous and novel variants in the X-linked *OFD1* gene. Pathogenic variants in the *OFD1* gene are associated with the X-linked recessive Joubert syndrome 10 and Simpson-Golabi-Behmel syndrome, type 2 as well as the X-linked dominant Orofaciodigital syndrome I (21). *OFD1* variants have previously been reported in association with inherited renal cystic disease, with hypertension (22). Sharma et al. described an XY male with a partial deletion of the *OFD1* gene associated with hypogonadism (23).

Non-synonymous, heterozygous rare and novel variants in the *GNRH1, GNRHR*, and *GLI3/INSR* genes were observed in case 14. She was seen 6.5 years for abundant vaginal bleeding. This was immediately preceded by a rapid breast development (B3-4 Tanner stage). Ultrasonography showed a pubertal uterus with a right OC measuring 10 mm. Rare biallelic and heterozygous variants in the *GNRH1* gene are associated with HH-12 with or without anosmia (24). Patients carrying heterozygous *GNRH1* variants usually have additional variants in other genes known to cause HH (25). This is the situation with this girl who, in addition to the rare p.E51D GNRH1 missense variant, carries a rare heterozygous p.Q106R variant in the *GNRHR* gene. The highly conserved GNRH1 p.E51 residue variant is located within GnRH-associated peptide (GAP) and it is predicted to be damaging by PolyPhen2. The p.Q106R change is a well-characterized GNRHR pathogenic variant, which is a known and a relativity common cause of HH (25). Most cases of HH that are caused by *GNRHR* variants, are biallelic, consistent with an autosomal recessive mode of inheritance. However, digenic inheritance of HH has also been reported due to a monoallelic *GNRHR* variant together with monoallelic variants in other genes causing HH including *PROKR2, FGFR1*, and *WDR11* (26–28). Basal plasma concentrations of LH and FSH were low and did not increase after GnRH test, while the plasma estradiol concentration was prepubertal. The hypothalamic-pituitary region was normal on magnetic resonance imaging. The girl also carried rare heterozygous *GLI3* and *INSR* non-synonymous variants. *GLI3* variants have been reported in association with PP with prolonged vaginal bleeding (29).

The mother of case 15 had a history of prepubertal OC with no evidence of PP. Her brother began his puberty at the lower range of normal, with testicular volume at 7 mL at 10 years. She was referred for breast development and a clear vaginal discharge at 6.6 years. Basal plasma concentrations of LH and FSH were low and did not increase after GnRH test, while the estradiol concentration was at 576 pmol/l. She was treated with Cyproterone Acetate for a total of 3.5 years at an initial dose of 50 mg/day because the high plasma estradiol concentration and large OC. Three months later the OC disappeared, the breast development and the plasma estradiol concentration decreased (<37 pmol/l) leading to decrease the dose to 12.25 mg/day. Menarche occurred at 12 years, followed by regular menstruations at the last evaluation at 13 years. She carried a novel non-synonymous variant in the *CDON* gene, p.V370A, which is predicted to be possibly damaging. Heterozygous variants in *CDON* are associated with a range of phenotypes from holoprosencephaly to pituitary stalk interruption syndrome (30, 31). The girl also carries a rare heterozygous missense variant in the *CHD7* gene. Heterozygous variants in *CHD7* are associated with autosomal dominant CHARGE syndrome or HH-5 with or without anosmia. The latter is considered to reflect a mild presentation of the CHARGE syndrome (32).

Case 17 carried known pathogenic and potentially pathogenic variants in the *RNF216* and *PROKR2* genes. She was seen at 8.8 years for vaginal bleeding associated with breast and pubic hair developments. Ultrasonography showed many cysts ≥10 mm. The gonadotropins response to the GnRH test was prepubertal. The PROKR2 p.R85C variant carried by the affected girl has been previously reported to cause of a mild form of autosomal dominant HH (33, 34). Interestingly some inactive PROKR2 mutants have also been reported to cause PP presumably by enhancing the functional property of coexisting wild-type proteins (35). This suggests a mechanism of how these variants could potentially lead to the phenotype. As well as the PROKR2 pathogenic variant, patient 17 also carries a potentially pathogenic, rare heterozygous missense variant in the *RNF216* gene. Homozygous or compound heterozygous mutation in the *RNF216* gene cause the Gordon Holmes syndrome with HH (36, 37). It cannot be excluded that in this case the phenotype is caused by a combination of variants in the *PROKR2* and *RNF216* genes. Case 1 also carries a nonsense variant in the *PNPLA6* gene that is associated with Gordon Holmes syndrome.

Case 18 was referred for vaginal bleeding at 9.2 years. There was no breast or pubic hair development. The gonadotropins response to the GnRH test and plasma estradiol concentration were prepubertal. The following 2 years were characterized by periodic vaginal bleeding, once a month and then twice a month, until 12 years when regular menstruations occurred. Iterative pelvic ultrasonographies did not reveal any OC but indirect evidence for OC was seen by a blood effusion in the Douglas cul-de-sac. This girl carried a rare non-synonymous variant in the *GLI3* and a novel non-synonymous variant in the *LEPR* gene. The clinical significance of the GLI3 p.M1312R variant is unclear, although as mentioned previously variants in this factor have been associated with PP (29). The LEPR p.P910S variant is predicted to by pathogenic by all *in silico* tools. Biallelic variants in the LEPR gene causes obesity and pituitary dysfunction leading to HH (38, 39). However, this girl did not present with obesity nor clinical evidence of pituitary dysfunction.

A comparison of all of the 18 exome datasets was performed for genes other than those involved in HH, which carry rare or novel potentially pathogenic variants that could contribute to the phenotype. However, rare/novel and potentially pathogenic variants in genes shared between 2 or more individuals was not observed.

## Strengths and Limitations of the Study

A strength of this study is that we performed a homogeneous and comparable evaluation and exome study in a well-characterized cohort of girls with isolated OC. The evaluation criteria were quantitative. Furthermore, this cohort of OC patients, although small, represents one of the largest reported to date to our knowledge.

This study also has limitations. This was a retrospective study with a small sample size although the use of a single investigator limited the inherent bias. Clinical and radiological were performed to exclude McCune-Albright syndrome but not in all patients and in the OC liquid collected at surgery only in case 13. However, the exome data excluded pathogenic variants in *GNAS*.

Although there was no history of personal or familial anosmia, no olfactive testing was performed. GnRH testing was not performed in 2 girls with prenatal OC. The control group for the genetic data consisted of individuals from the GnomAD database, however, there is no information on puberty or presence of ovarian cysts in this database. Functional studies were not performed on genetic variants. Although some girls were followed up until 13 years of age, there is not a long term follow up to fully determine the impact of variants involving HH gene, such as LH/FSH secretion and time of puberty and fertility. We cannot formally exclude that environmental factors may contribute to the phenotype.

## Conclusions

Of the 18 girls studied here 9 carried rare or novel variants in genes associated with HH. Of these 9 girls, 3 harbored gene variants that have been previously published as a cause of HH. This suggests that variants in genes associated with HH may contribute to the etiology of OC. Eight of 16 girls had a basal plasma concentrations of gonadotropins which were undetectable and did not increase after GnRH test. Five of these 8 also had high levels of estradiol suggesting a retrocontrol effect but 3 had prepubertal concentrations. These data reflect previous studies which indicated that the majority of girls with precocious pseudopuberty associated with an OC had basal plasma concentrations of LH and FSH that are either low or undetectable and that do not increase after the GnRH test, whilst the others had a prepubertal response (4, 5). The occurrence of regular menstruations in two of the patients needs to be further monitored in the long term. The potential contribution of each variant to the phenotype cannot be established in such a small cohort. The mechanism involved remains unknown. Gonadotropin-independent precocious puberty due to the presence of estrogen-secreting OC suggests an isolated autonomous ovarian disease. Here, we identified several known or potentially pathogenic variants in genes known to cause HH. However, pathogenic variants in genes causing hypogonadotropic pubertal delay such as the kisspeptins, tachykinins and their receptors are considered to be due to a failure of gonadotropin secretion by the anterior pituitary (40, 41). It is not at first obvious to reconcile these two observations. However, several recent studies have shown that kisspeptins, tachykinins and their receptors are locally synthesized in cumulus cells and mural granulosa cells of healthy women (42–44). Although kisspeptin can induce ovulation and trigger egg maturation the exact physiological role of these factors in the ovary has still not been established. Recently, a reduced expression of *TAC3* and *TAC3R* was observed in cumulus cells and mural granulosa cells from polycystic ovary syndrome (PCOS) patients (45). This lead the authors to suggest that altered expression of kisspeptins and tachykinins in the supporting cells of the ovary could play a role in the pathophysiology of PCOS. Although a similar mechanism may be involved in the development of ovarian cysts in our cohort we wish to stress that additional analysis on independent cohorts of OC need to be performed and also to exclude that these HH variants are a co-incidental finding in these cases.

## Data Availability Statement

The data presented in the study are deposited in the ClinVar (https://www.ncbi.nlm.nih.gov/clinvar/) repository, accession numbers (VCV001064513, VCV001064507, VCV001064517, VCV001064514, VCV001064506 and VCV001064515). This data is publicly available.

## Ethics Statement

The studies involving human participants were reviewed and approved by Comité de Protection des Personnes Ile de France III, FORDEV 3445. Written informed consent to participate in this study was provided by the participants' legal guardian/next of kin. All clinical investigations were conducted according to the principals outlined in the Declaration of Helsinki. Written, informed consent was given by all the parents for the clinical-biological evaluation (included in their hospital medical record) and for the molecular biology analyses with the use of protocols approved by local and national Ethics Committees (Comité de Protection des Personnes Ile de France III, FORDEV 3445). With the exception of routine patient care, no other interventions were performed as part of the study. Informed consent was obtained from the [individual(s) AND/OR minor(s) legal guardian/next or kin] for the publication of any potentially identifiable images or data included in this article. The authors had no direct interaction with the patients enrolled in the study except during their medical follow-up, which was performed by Raja Brauner. Patient information was anonymized by the medical secretary prior to analysis.

## Author Contributions

RB conceived the study, oversaw the design and study coordination, carried out participant recruitment, and wrote the original draft. JB-T performed formal analysis. AB performed data curation. KM directed the exome sequencing data and wrote the final version of the manuscript. All authors read and approved the final manuscript.

## Conflict of Interest

The authors declare that the research was conducted in the absence of any commercial or financial relationships that could be construed as a potential conflict of interest.

## Abbreviations

CV: coefficient of variation
FSH: Follicle-stimulating hormone
GnRH: Gonadotropin-releasing hormone
HH: Hypogonadotropic hypogonadism
LH: Luteinizing hormone
OC: Isolated ovarian cyst
PCOS: polycystic ovary syndrome
PP: Precocious puberty.
